# Supplementary material for: Gene interaction perturbation network deciphers a high-resolution taxonomy in colorectal cancer
Source: eLife. 2022 Nov 8;11:e81114. doi: 10.7554/eLife.81114 (PMC9643007; doi:10.7554/eLife.81114)
Supplement: Supplementary file 16. [file elife-81114-supp16.docx]

**Supplementary methods**

**Publicly available data generation and processing**

***Human CRC bulk cohorts*.** The integrated discovery cohort contained 2,167 patients with CRC from 19 independent datasets who fulfilled the following criteria: (1) available expression profiles obtained using the same chip platform (Affymetrix Human Genome U133 Plus 2.0 Array, GPL570) with raw CEL data; (2) overall survival (OS), relapse-free survival (RFS), fluorouracil-based adjuvant chemotherapy (ACT) or bevacizumab treatment information available. Another 19 independent datasets comprised 3,420 patients with CRC from different platforms and sequencing techniques (microarray or RNA-seq) were used for validation.

***Normal tissue data.*** The Genotype-Tissue Expression (GTEx, <https://gtexportal.org>) project has established a tissue biobank with all samples from normal tissues rather than paracancerous tissues (e.g., TCGA). The RNA-seq data of 308 normal colorectal samples were obtained from the GTEx portal.

***Immunotherapy cohorts*.** Three datasets (n =414) with immunotherapeutic annotations and expression profiles were derived from the following studies: Hugo and colleagues(***Hugo et al.,* *2016***) (GSE78220, n =27), Rose and colleagues(***Rose et al.,* *2021***) (GSE176307, n =89) and Mariathasan and colleagues(***Mariathasan et al.,* *2018***) (IMvigor210, n =298). Patients in Hugo cohort were treated with *PD-1* blockade, patients in Rose cohort were treated with *PD-1*/*PD-L1* blockade, and patients in Mariathasan cohort were treated with *PD-L1* blockade. All patients were performed with next-generation sequencing.

***Multi-omics data for TCGA-CRC*.** The TCGA-CRC multi-omics data, including RNA-seq (raw count), proteome (Reverse Phase Protein Array), HumanMethylation450 array, whole-exome sequencing (VarScan MAF files), and copy number variation (CNV) data, were derived from TCGA portal (<https://portal.gdc.cancer.gov>). The neoantigen load (NAL) and homologous recombination deficiency (HRD) score measured by TCGA officials were available in TCGA pan-cancer atlas(***Thorsson et al.,* *2018***).

***CRC cell lines*.** The Cancer Cell Line Encyclopedia (<https://sites.broadinstitute.org/ccle>, CCLE) stores the processed multi-omics data of cancer cell lines. We retrieved 55 CRC cell lines with both transcriptome and metabolomics data (including 225 metabolites) for further explorations(***Li et al.,* *2019***).

***PDX model data.*** Two CRC PDX datasets were generated from two previous studies(***Isella et al.,* *2017; Uronis et al.,* *2012***). Uronis cohort (chip data) encompassed 27 matched human primary CRC and mouse xenografts and Isella cohort (RNA-seq data) included 116 matched liver metastatic CRC and mouse xenografts.

***Radiotherapy cohort.*** The GSE56699 comprises 72 primary rectal cancer with radiotherapeutic annotations, including 11 pairs of preoperative radiotherapy specimens and matched post-treatment surgical specimens(***Isella et al.,* *2015***).

***Single-cell RNA sequencing data.*** A total of 1,591 single cells from 11 patients with CRC using Fluidigm based single-cell RNA-seq protocol were achieved from Li et al. study(***Li et al.,* *2017***). The cell annotations were obtained from the reference component analysis (RCA).

***Pharmacogenomic datasets.*** Drug response and molecular data of human cancer cell lines were generated from the Cancer Therapeutics Response Portal (CTRP, <https://portals.broadinstitute.org/ctrp>), Profiling Relative Inhibition Simultaneously in Mixtures (PRISM, [https://depmap.org/portal/prism](https://depmap.org/portal/prism/)), and Genomics of Drug Sensitivity in Cancer (GDSC, [https://www.cancerrxgene.org](https://www.cancerrxgene.org/)) datasets. The area under the dose-response curve (AUC) values measured drug sensitivity (the AUC values in CTRP range from 0 to 30, in PRISM and GDSC range from 0 to 1). AUC values were log-transformed, with lower values indicating increased sensitivity to treatment. Drugs with missing AUC values in more than 20% of cell lines were removed, and the remaining missing values were imputed using the k-nearest neighbors (KNN) imputation procedure. The hematopoietic and lymphoid tissue-derived cell lines were excluded. Ultimately, CTRP contains the response data for 481 drugs over 835 cell lines, PRISM contains the response data for 1,448 drugs over 482 cell lines, and GDSC contains the response data for 265 drugs over 828 cell lines.

***Data processing.*** Published microarray data were obtained from the Gene Expression Omnibus (GEO, <https://www.ncbi.nlm.nih.gov/geo>). The robust multiarray average (RMA) preprocessing and normalization of raw CEL files from Affymetrix GeneChip® arrays were performed via *affy* package. Gene expression presence and absence were measured via the barcode algorithm(***McCall et al.,* *2011***), and genes not presented in all samples were discarded. To remove potential multicenter batch effects of discovery cohort, data were corrected using ComBat approach implemented in *sva* package (Figure S1)(***Leek et al.,* *2012***). For Illumina and Agilent microarrays, the processed data were obtained from the authors. The RNA-seq data (raw count) from TCGA-CRC and GTEx were converted to transcripts per kilobase million (TPM) and further log_2_ transformed. For somatic mutation data, somatic variants were detected using TCGA VarScan2 pipeline. A binary mutation profile was used to indicate the presence or absence of a gene mutation, and then gene-level mutations were generated after filtering out the synonymous mutations. For CNV data, segmented CNV values were generated by circular binary segmentation algorithm implemented in *DNAcopy* package. Then, all genes were mapped onto this segmented data to obtain gene-level and broad-level CNV values using GISTIC2.0. For DNA methylation data, we firstly excluded probes with >20% of missing values across samples and the probes for sex chromosomes, and the remaining missing values were imputed using the KNN imputation procedure. Then, each gene with the average beta value of the promoter region probes was assigned the final methylation values. For drug outcomes, the RECIST guideline (version 1.1)(***Eisenhauer et al.,* *2009***) indicates treatment responders and non-responders are defined as having a complete response (CR)/partial response (PR) and stable disease (SD)/progressive disease (PD), respectively.

**Description of previous CRC classifications**

To compare our subtypes with previously reported CRC classifications, the discovery cohort was reclassified according to the previous subtype criteria, including consensus molecular subtypes (CMS)(***Guinney et al.,* *2015***), CRC intrinsic subtypes (CRIS)(***Isella et al.,* *2017***), colon cancer subtypes (CCS)(***De Sousa et al.,* *2013***), CRCAssigner (CRCA)(***Sadanandam et al.,* *2013***), and Cartes d’Identité des Tumeurs (CIT)(***Marisa et al.,* *2013***), respectively. CMS integrated six independent classification systems into four CRC groups (CMS1-4) based on gene transcription and are prognostic in early-stage and first-line metastatic settings. Isella et al. deployed human-specific expression profiling of CRC PDXs to assess cancer-cell intrinsic transcriptional features, and identified five CRIS subtypes (CRISA-E). CCS was developed using a previously developed unsupervised consensus-based clustering technique, and formed three clusters generated the most robust classification (CCS1-3). Sadanandam et al. discovered five CRCA subtypes (CRCA1-5) shared similarities to distinct cell types within the normal colon crypt and showed differing degrees of ‘stemness’ and Wnt signaling. The French national CIT program enrolled a multicenter cohort of 750 patients with stage I to IV CRC and found six CIT subtypes (CIT1) based on cluster expression centroid classification.

**Background network**

The gene interaction-perturbation pipeline applies the discovery cohort as the tumor sample input and the GTEx cohort as the normal sample input. A pathway-derived analysis requires constructing the protein interaction functional networks projected by candidate genes(***Chen et al.,* *2021***). Data from the Reactome Pathway Database (<https://reactome.org>) focus on the reaction, which was utilized to establish the biological interaction networks(***Jassal et al.,* *2020***). Network nodes that absented in our cohorts were removed and existing nodes were integrated into the large background network with 148,942 interactions in total.

**Interaction-perturbation-based program**

The flowchart of this program is shown in Figure 1. For each gene within the background network, we first calculated its rank in individuals based on its gene expression value in each sample (the smallest expression value corresponds to the minimum rank, and the largest expression value corresponds to the maximum rank). Thus, the expression matrix was converted into the rank matrix by ranking all genes according to the expression values in all samples. The *R_a,s_* represents the rank of gene *G_a_* in sample *s*. Subsequently, the delta rank matrix, which rows and columns denoted interactions in the background network and samples, is generated from the rank matrix. For instance, a delta rank (represented by *δ_i,s_*) was calculated by subtracting the ranks for each gene pair (e.g., *G_a_* and *G_b_*) connected by an interaction in the background network, as follows:

$$\delta_{i,s}=R_{a,s}-R_{b,s}$$

Where genes *G_a_* and *G_b_* are connected by interaction *i* in the background network.

Gene interactions are highly conservative within normal samples, and interaction perturbations are rare(***Sahni et al.,* *2015***). The within-sample delta ranks of gene pairs are highly stable among samples under normal conditions but are often widely disrupted after certain treatments, such as gene knockdown, gene transfection, drug treatment and tissue canceration(***Li et al.,* *2019***). Hence, we hypothesized that the background network is stable across all normal samples, and then the interactions within normal samples served as the benchmark network. We ranked genes according to their mean gene expression value among normal samples and similarly calculated the delta rank as the benchmark delta rank vector with elements denoted by $\bar{\delta_{i}}$, where *i* is an interaction in the background network. This vector measures the mean relative ranks of gene pairs in all normal samples. Gene interactions in each patient should be compared with the benchmark network, and the corresponding difference represents the gene interaction perturbation for that patient. Upon subtracting the benchmark delta rank vector from the delta rank of each sample, we finally obtained the interaction-perturbation matrix $\Delta$ with element $\Delta_{is}$. For an interaction *i* in the background network and an individual sample s,

$\Delta_{is}=\delta_{i,s}-\bar{\delta_{i}}$.

The interaction-perturbation matrix can measure the sample-specific interaction perturbation in the same whole background network effectively. Each column of the interaction-perturbation matrix represents the gene interaction perturbations for an individual sample, i.e., the sample-specific perturbation of the gene interaction.

**Discovery of the gene interaction network-based subtypes**

The subtypes were deciphered in the discovery cohort using the consensus clustering approach that required two elements, including features in rows and samples in columns. The clustering features needed to meet two aspects: (1) being able to significantly distinguish tumor from normal samples; (2) maintaining strong heterogeneity within all tumor samples with high variability. First, the differential analysis between tumor and normal samples for each interaction was performed. The top 30,000 interactions (approximately 20%) that differed significantly between tumor and normal samples and the top 30,000 interactions with high standard deviation (SD) among all CRC samples were selected. Subsequently, the intersection of two sets with 30,000 gene interactions overall CRC samples were retained for clustering work.

Furthermore, the perturbation matrix with the selected interactions was subjected to the consensus clustering procedure(***Wilkerson and Hayes,* *2010***), a resampling-based clustering algorithm implemented in the *ConsensusClusterPlus* package(***Wilkerson and Hayes,* *2010***). This procedure using the partitioning around medoids approach and 1-Spearman correlation distance was performed 5000 iterations on 80% of interactions and 80% of samples selected randomly. All derived partitions for a cluster *K* (2-10) were summarized by clustering the (samples $\times$ samples) co-classification matrix. Subsequently, the consensus score matrix, cumulative distribution function (CDF) curve, proportion of ambiguous clustering (PAC) score, and Nbclust were synthetically used to determine the optimal number of clusters. A higher consensus score between two samples indicates they are more likely to be grouped into the same cluster in different iterations. The consensus values range from 0 (never clustered together) to 1 (always clustered together) marked by white to dark brown. In the CDF curve of a consensus matrix, the lower left portion represents sample pairs rarely clustered together, the upper right portion represents those almost always clustered together, whereas the middle segment represents those with ambiguous assignments in different clustering runs. The proportion of ambiguous clustering (PAC) measure quantifies this middle segment; and is defined as the fraction of sample pairs with consensus indices falling in the interval (u1, u2) ∈ [0, 1] where u1 is a value close to 0 and u2 is a value close to 1 (for instance u1=0.1 and u2=0.9). A low value of PAC indicates a flat middle segment, and a low rate of discordant assignments across permuted clustering runs. PAC for each K is CDF_k_(u2) - CDF_k_(u1)(***Șenbabaoğlu et al.,* *2014***). According to his criterion, we can therefore infer the optimal number of clusters by the K value having the lowest PAC. The Nbclust uses 26 mathematic criteria to select the optimal number.

**Generation of the GINS classifier**

To identify GINS subtypes in novel datasets using a small list of genes, we developed a gene expression-based classifier in three steps. First, our analysis included only samples that statistically belonged to the core of each subtype. Excluding samples with negative silhouette width has been shown to minimize the impact of sample outliers on the identification of subtype markers, as described in TCGA glioblastoma classification(***Verhaak et al.,* *2010***). Second, the significance analysis of microarrays (SAM)(***Tusher et al.,* *2001***) was employed to identify 762 genes significantly differentially expressed across the GINS subtypes. This is a well-established method that looks for large differential gene expression relative to the spread of expression across all genes. Sample permutation is used to estimate false discovery rates (FDR) associated with sets of genes identified as differentially expressed. The threshold was set to Benjamini–Hochberg-corrected FDR <0.0001. Third, the differentially expressed genes were further trained by prediction analysis for microarrays (PAM)(***Tibshirani et al.,* *2002***) to build a classifier. The PAM eliminated the contribution of genes that differentially express below a specific threshold, ΔPAM, relative to the subtype-specific centroids. The leave-one-out cross-validation (LOOCV) was performed to ultimately identify 289 genes that had the lowest misclassification error (1.8%). Using this strategy, a centroid-based classifier was built, and six centroids were computed on the gene-median centered discovery cohort. For each validation dataset, the distance to the six centroids of each sample was computed and samples were assigned to the closest centroid subtype. As previously reported(***Marisa et al.,* *2013***), the decision rule was based on the diagonal quadratic discriminant analysis method (DQDA) is defined as follows:

$$DQDA\left( X \right)=\underset{j\in\left\{ GINS1-6 \right\}}{Arg min} \left( \sum_{i=1}^{N} \frac{\left( x_{i}-u_{j,i} \right)^{2}}{v_{j,i}} \right)+\sum_{i=1}^{N} log(v_{j,i})$$

where *N* is the number of genes, *x* represents the expression normalized values, $u_{j,i}$ and $v_{j,i}$ were denoted by the mean and the variance of the gene *i* across samples of the subtype *j* from the discovery cohort. The classification confidence was assessed by identifying outliers (too distant samples) and mixed assignment samples (when a sample is close to several centroids). More specifically, a sample was defined as an outlier if its distance to the nearest centroid is superior to *n* times the median absolute deviation (MAD) of the distances of the samples used to compute the centroid; *n* is defined as the maximum (distances to centroid-median_distances to centroid_)/MAD_distances to centroid_. A sample has a mixed assignment if the difference of its distance to centroid is inferior to the 1st decile of the difference between centroids on data used to compute centroids.

**Human tissue specimens**

The human cancer tissues used in this study were approved by the Ethics Committee of The First Affiliated Hospital of Zhengzhou University. Overall, 214 frozen surgically resected CRC tissues were collected from The First Affiliated Hospital of Zhengzhou University. All patients provided written informed consent; received available standard systemic therapies; were aged 18 years or older; had adequate hematologic, renal, and liver function; had Eastern Cooperative Oncology Group performance status of 0 or 1; and had measurable disease according to Response Evaluation Criteria in Solid Tumours (RECIST, version 1.1)(***Eisenhauer et al.,* *2009***). Responders and nonresponders were defined as having a complete response (CR)/partial response (PR) and stable disease (SD)/progressive disease (PD), respectively. Detailed baseline data of patients with CRC are displayed in Supplementary file 15.

**RNA preparation and Quantitative Real-Time PCR (qRT-PCR)**

Total RNA was isolated from CRC tissues using RNAiso Plus reagent RNA quality was evaluated using a NanoDrop One C (Waltham, MA, USA), and RNA integrity was assessed using agarose gel electrophoresis. An aliquot of 1 μg of total RNA was reverse transcribed into complementary DNA (cDNA) according to the manufacturer's protocol using a High-Capacity cDNA Reverse Transcription kit (TaKaRa BIO, Japan). qRT-PCR was performed using SYBR Assay I Low ROX (Eurogentec, USA) and SYBR® Green PCR Master Mix (Yeason, Shanghai, China) to detect the expression of 14 genes. The expression value was normalized to *GAPDH*, and then log2 transformed for subsequent analysis. The primer sequences of the included 14 genes and *GAPDH* were shown in Supplementary file 4.

**Quantitative PCR miniclassifier**

To facilitate translation of the GINS subtypes in clinical settings, we intended to develop a quantitative PCR (qPCR) miniclassifier and further validate our subtypes in 214 clinical CRC samples from The First Affiliated Hospital of Zhengzhou University. Using 289 genes from the PAM classifier, we proposed a six-step pipeline to develop the simplified GINS miniclassifier in the discovery cohort:

1. For a gene, we extracted the expression of this gene in each subtype, and if the expression of one subtype was significantly higher than that of all other subtypes, this gene is defined as a specific gene of this subtype. In total, we identified 191 subtype-specific genes.
2. Bootstrapping method was further used to test these genes (from the first step). We extracted 70% of samples randomly from the entire cohort and performed univariate Logistic regression analysis on these samples to assess the correlation between the gene expression and prognosis. This procedure was repeated 1000 times and 93 genes that were incorporated in all resample runs (achieved P < 0.05 in robustness testing) were kept for next step analysis.
3. To further select genes with the most information, the LASSO regression was applied for further dimension reduction. The LASSO algorithm is a popular method for regression with high-dimensional predictors. It introduces a penalty parameter λ to shrink some regression coefficients to exactly zero. The penalty parameter λ, called the tuning parameter, controls the amount of shrinkage: the larger the value of λ, the fewer the number of predictors selected. The 10-fold cross-validation was used to determine the optimal values of λ via the one-standard-error rule. The optimal λ is the largest value for which the deviance is within one standard-error of the smallest value of deviance. In this process, 14 key genes were retained.
4. The discovery cohort was firstly divided into the training (70%) and testing (30%) datasets. With the expression profiles of 14 key genes, the random forest was employed to build a classifier with the following criteria: Gini impurity, 1,000 trees, minimal nodesize = 3, bootstrap sample, and 10-fold cross-validation. The random forest analysis was repeated 1,000 times, and the model with the smallest of out-of-bag (OOB) error rate was considered as the optimal classifier.
5. The five indicators including accuracy, precision, recall, F1-score, and specificity were utilized to evaluate the performance of our miniclassifier in both training and testing datasets.

**Gene set enrichment analysis**

The *clusterProfiler* package(***Yu et al.,* *2012***) was utilized to perform downloaded from the gene set enrichment analysis (GSEA). The significance of enrichment was estimated using default settings and 1,000 gene permutations.

**Gene set variation analysis**

The gene set variation analysis (GSVA) was conducted via the *GSVA* package(***Hanzelmann et al.,* *2013***). GSVA is a non-parametric, unsupervised method for estimating variation of gene set enrichment through the samples of an expression data set. GSVA performs a change in coordinate systems, transforming the data from a gene by sample matrix to a gene-set by sample matrix, thereby allowing the evaluation of pathway enrichment for each sample.

**Sample set enrichment analysis**

To assess the extent to which six subtypes captured samples with stronger transcriptional signatures, we introduced a framework termed ‘Sample Set Enrichment Analysis’ (SSEA)(***Isella et al.,* *2017***). In SSEA, all samples are ranked by the integrated scores, and the ranked sample list was further subjected to the GSEA procedure to test whether the ‘sample set’ for each subtype enriches high-ranking samples. As previously reported(***Isella et al.,* *2017***), a signature score was computed for each signature and each sample, as follows:

$${Score}_{i}=\bar{{Cor}^{+}}-\bar{{Cor}^{-}}$$

where $\bar{{Cor}^{+}}$ is the average expression value of the genes positively correlated with the subtype and $\bar{{Cor}^{-}}$ is the average expression value of the genes negatively correlated with the subtype in sample *i*.

For SSEA of mitogenic/anti-apoptotic autocrine loops(***Isella et al.,* *2017***), a total of 472 receptor/ligand interactions were downloaded from the Database of Ligand Receptor Partners (<http://dip.doembi.ucla.edu/dip/DLRP.cgi>). All samples in SSEA framework were ranked according to ‘receptor activation index’, which were computed by averaging the expression of receptor and its ligands, as follows:

$${Receptor activation index}_{i}=\frac{Receptor+\bar{Ligands}}{2}$$

where *Receptor* is the mRNA expression of the receptor of interest and $\bar{Ligands}$ stands for the average expression of its corresponding ligands in sample *i*.

**Cancer-immunity cycle**

Based on the rationale that immunity within tumors is a dynamic process, Karasaki and colleagues(***Karasaki et al.,* *2017***) have proposed an immunogram for the cancer-immunity cycle (CIC) depicted by eight axes of the immunogram score (IGS): IGS1, T cell immunity; IGS2, tumor antigenicity; IGS3, priming and activation (activated dendric cell enrichment); IGS4, trafficking and infiltration; IGS5, recognition of tumor cells; IGS6, inhibitory cells; IGS7, checkpoint expression; and IGS8, inhibitory molecules. The gene sets of IGS1-IGS8 were retrieved from a previous study(***Karasaki et al.,* *2017***). The single‐sample gene‐set enrichment analysis (ssGSEA) approach was leveraged to measure the IGS, and the immunogram radar displayed the normalized IGS value of six subtypes.

**Assessment of immunotherapeutic potential**

To predict the putative response to immune checkpoint blockade (ICB), CRC samples were scored using T-cell inflammatory signature (TIS) and Tumor Immune Dysfunction and Exclusion (TIDE) approaches. TIS proposed by Ayers et al. was used to predict clinical response to PD-1 blockade. The signature was composed of 18 inflammatory genes associated with antigen presentation, chemokine expression, cytotoxic activity, and adaptive immune resistance(***Ayers et al.,* *2017***). The TIDE algorithm (<http://tide.dfci.harvard.edu/>) integrates the expression signature of two primary mechanisms of immune evasions: T cell dysfunction and T cell exclusion, to model tumor immune evasion. Patients with higher TIDE score suggest the greater potential of tumor immune evasion; thus, these patients would derive worse immunotherapy response(***Jiang et al.,* *2018***). The immunophenoscore (IPS) was applied to assess the immune state of each sample. IPS is a scoring scheme that quantifies the immunogenicity of tumor samples using a variety of markers of immune response or immune tolerance. The higher the IPS z-score, the stronger the immunogenicity and immunotherapeutic potential of the sample(***Charoentong et al.,* *2017***).

**Characteristics of multi-omics alterations**

For mutation analysis, using the VarScan pipeline and MutSigCV algorithm(***Lawrence et al.,* *2013***), we retained genes with mutation frequency >10% and MutSigCV q-value <0.05. For copy number variation (CNV) analysis, the DNAcopy pipeline used Affymetrix SNP 6.0 array data to identify genomic regions that are repeated and infer the copy number of these repeats. The copy number values were further transformed into segment mean values, which were represented as log2 (copy-number/2). With -0.3 and 0.3 as cut-off points, genes were marked as deletion (<-0.3), neutral (-0.3~0.3), and amplification (>0.3). GISTIC 2.0 software(***Mermel et al.,* *2011***) was applied to define the recurrently amplified and deleted regions. Subsequently, we retained focal segments with CNV frequency >40% and GISTIC q-value <0.05. For methylation profile analysis, we first assigned DNA methylation values for each gene with the average beta value of the probes mapped to the promoter region, including TSS200 (region from –200 bp upstream to the transcription start site (TSS) itself), 1stExon (the first exon), TSS1500 (from –200 to –1500 bp upstream of TSS), and 5′UTR in order. For each phenotype, we identified the epigenetically silenced genes (ESGs) using the following criteria: (1) excluding the CpG sites methylated in normal tissues (mean β-value of > 0.2) or less than 10% of the tumor samples; (2) the DNA methylation data was divided into the methylation group and unmethylation group, according to the cutoff (β-value = 0.3); (3) for each gene, if the difference between the corresponding gene mean expression in the unmethylated group and that in the methylated group was > 1.64 standard deviations of the unmethylated group, the gene would be labeled as epigenetically silenced(***Liu et al.,* *2021***).

***Assessments of TMB, NAL, and CNV burden***

TMB was defined as the total non-silent somatic mutation counts in coding regions, encompassing missense, nonsense, frameshift insertion, frameshift deletion, in-frame insertion, in-frame deletion, and splice site mutation. The “maftools” R package was utilized to process mutation data and calculate the TMB of each patient. Neoantigens of the TCGA-CRC dataset measured by TCGA official were available in TCIA database (<https://tcia.at/neoantigens>)(***Charoentong et al.,* *2017***). The fraction of genome alteration (FGA), fraction of genome gained (FGG), and fraction of genome lost (FGL), were defined as the ratio of total CNV/gain/lost bases to all bases, respectively. The TCGA-CRC cohort was calculated based on copy number segment data as follows:

$$FGA=\frac{Altered bases}{Total bases}$$

$$FGG=\frac{Gianed bases}{Total bases}$$

$$FGL=\frac{Loss bases}{Total bases}$$

**Estimating drug sensitivity in clinical cohort**

To facilitate the subtype-based targeted interventions, we introduced an integrated pipeline to identify potential therapeutic agents for each subtype(***Yang et al.,* *2021***) (Figure S20A). Three pharmacogenomic datasets, CTRP, PRISM, and GDSC, store large-scale drug response and molecular data of human cancer cell lines, enabling accurate prediction of drug response in clinical samples(***Yang et al.,* *2021***). As mentioned above, stromal components could obscure the expression patterns of cancer cells in clinical samples. A purification algorithm termed *ISOpure*(***Quon et al.,* *2013***) was adopted to eliminate the contamination of stromal signal in the discovery cohort prior to conducting drug response prediction, and further yielded a purified tumor expression profiles comparable to cell lines(***Yang et al.,* *2021***). After purification, the proportion of stromal-rich subtypes (e.g., GINS2 and GINS4) were obviously decreased, suggesting the impact of stroma components had been eliminated (Figure S20B). A PDX dataset, GSE73255(***Isella et al.,* *2017***), is naturally uncontaminated by human stromal components. Hence, we tested our pipeline in the discovery cohort and GSE73255, and ultimately identified intersecting subtype-specific agents in two datasets. As previously reported(***Yang et al.,* *2021***), the model used for predicting drug response was ridge regression model implemented in the *pRRophetic* package(***Geeleher et al.,* *2014***). This predictive model was trained on mRNA expression profiles and drug response data of cancer cell lines with a satisfied predictive accuracy were evaluated by default 10-fold cross-validation, thus allowing the estimation of clinical drug response using only patients’ baseline gene expression data. We inputted the purified tumor expression profiles into the regression model to estimate the drug response of clinical samples, and ultimately determined the consensus agents in both the discovery cohort and GSE73255.

**Statistical analysis**

All data processing, statistical analysis, and plotting were conducted in R 4.0.5 software. Correlations between two continuous variables were assessed via Pearson’s correlation coefficients. The chi-squared or fisher exact test was applied to compare categorical variables, and continuous variables were compared through the Wilcoxon rank-sum test or T test. The coefficient of variation (CV) is the ratio of the standard deviation to the mean and shows the extent of variability in relation to the mean of the population. The higher the CV, the greater the dispersion. R was used to measure the fitting level of the power law curve, The better the curve fitting level is, the closer R is to -1. Kaplan-Meier analyses were performed via the survival package. All statistical tests were two-sided. *P* <0.05 was regarded as statistically significant.

**References**

Hugo W, Zaretsky JM, Sun L, Song C, Moreno BH, Hu-Lieskovan S, Berent-Maoz B, Pang J, Chmielowski B, Cherry G, Seja E, Lomeli S, Kong X, Kelley MC, Sosman JA, Johnson DB, Ribas A, Lo RS. 2016. Genomic and Transcriptomic Features of Response to Anti-PD-1 Therapy in Metastatic Melanoma *Cell* **165**:35-44. DOI: <https://doi.org/10.1016/j.cell.2016.02.065>, PMID: 26997480

Rose TL, Weir WH, Mayhew GM, Shibata Y, Eulitt P, Uronis JM, Zhou M, Nielsen M, Smith AB, Woods M, Hayward MC, Salazar AH, Milowsky MI, Wobker SE, McGinty K, Millburn MV, Eisner JR, Kim WY. 2021. Fibroblast growth factor receptor 3 alterations and response to immune checkpoint inhibition in metastatic urothelial cancer: a real world experience *Br J Cancer* **125**:1251-1260. DOI: <https://doi.org/10.1038/s41416-021-01488-6>, PMID: 34294892

Mariathasan S, Turley SJ, Nickles D, Castiglioni A, Yuen K, Wang Y, Kadel EE, III, Koeppen H, Astarita JL, Cubas R, Jhunjhunwala S, Banchereau R, Yang Y, Guan Y, Chalouni C, Ziai J, Senbabaoglu Y, Santoro S, Sheinson D, Hung J, et al. 2018. TGFbeta attenuates tumour response to PD-L1 blockade by contributing to exclusion of T cells *Nature* **554**:544-548. DOI: <https://doi.org/10.1038/nature25501>, PMID: 29443960

Thorsson V, Gibbs DL, Brown SD, Wolf D, Bortone DS, Ou Yang TH, Porta-Pardo E, Gao GF, Plaisier CL, Eddy JA, Ziv E, Culhane AC, Paull EO, Sivakumar IKA, Gentles AJ, Malhotra R, Farshidfar F, Colaprico A, Parker JS, Mose LE, et al. 2018. The Immune Landscape of Cancer *Immunity* **48**:812-830 e814. DOI: <https://doi.org/10.1016/j.immuni.2018.03.023>, PMID: 29628290

Li H, Ning S, Ghandi M, Kryukov GV, Gopal S, Deik A, Souza A, Pierce K, Keskula P, Hernandez D, Ann J, Shkoza D, Apfel V, Zou Y, Vazquez F, Barretina J, Pagliarini RA, Galli GG, Root DE, Hahn WC, et al. 2019. The landscape of cancer cell line metabolism *Nat Med* **25**:850-860. DOI: <https://doi.org/10.1038/s41591-019-0404-8>, PMID: 31068703

Isella C, Brundu F, Bellomo SE, Galimi F, Zanella E, Porporato R, Petti C, Fiori A, Orzan F, Senetta R, Boccaccio C, Ficarra E, Marchionni L, Trusolino L, Medico E, Bertotti A. 2017. Selective analysis of cancer-cell intrinsic transcriptional traits defines novel clinically relevant subtypes of colorectal cancer *Nat Commun* **8**:15107. DOI: <https://doi.org/10.1038/ncomms15107>, PMID: 28561063

Uronis JM, Osada T, McCall S, Yang XY, Mantyh C, Morse MA, Lyerly HK, Clary BM, Hsu DS. 2012. Histological and molecular evaluation of patient-derived colorectal cancer explants *PLoS One* **7**:e38422. DOI: <https://doi.org/10.1371/journal.pone.0038422>, PMID: 22675560

Isella C, Terrasi A, Bellomo SE, Petti C, Galatola G, Muratore A, Mellano A, Senetta R, Cassenti A, Sonetto C, Inghirami G, Trusolino L, Fekete Z, De Ridder M, Cassoni P, Storme G, Bertotti A, Medico E. 2015. Stromal contribution to the colorectal cancer transcriptome *Nat Genet* **47**:312-319. DOI: <https://doi.org/10.1038/ng.3224>, PMID: 25706627

Li H, Courtois ET, Sengupta D, Tan Y, Chen KH, Goh JJL, Kong SL, Chua C, Hon LK, Tan WS, Wong M, Choi PJ, Wee LJK, Hillmer AM, Tan IB, Robson P, Prabhakar S. 2017. Reference component analysis of single-cell transcriptomes elucidates cellular heterogeneity in human colorectal tumors *Nat Genet* **49**:708-718. DOI: <https://doi.org/10.1038/ng.3818>, PMID: 28319088

McCall MN, Uppal K, Jaffee HA, Zilliox MJ, Irizarry RA. 2011. The Gene Expression Barcode: leveraging public data repositories to begin cataloging the human and murine transcriptomes *Nucleic Acids Res* **39**:D1011-1015. DOI: <https://doi.org/10.1093/nar/gkq1259>, PMID: 21177656

Leek JT, Johnson WE, Parker HS, Jaffe AE, Storey JD. 2012. The sva package for removing batch effects and other unwanted variation in high-throughput experiments *Bioinformatics* **28**:882-883. DOI: <https://doi.org/10.1093/bioinformatics/bts034>, PMID: 22257669

Eisenhauer EA, Therasse P, Bogaerts J, Schwartz LH, Sargent D, Ford R, Dancey J, Arbuck S, Gwyther S, Mooney M, Rubinstein L, Shankar L, Dodd L, Kaplan R, Lacombe D, Verweij J. 2009. New response evaluation criteria in solid tumours: revised RECIST guideline (version 1.1) *Eur J Cancer* **45**:228-247. DOI: <https://doi.org/10.1016/j.ejca.2008.10.026>, PMID: 19097774

Chen Y, Gu Y, Hu Z, Sun X. 2021. Sample-specific perturbation of gene interactions identifies breast cancer subtypes *Brief Bioinform* **22**. DOI: <https://doi.org/10.1093/bib/bbaa268>, PMID: 33126248

Jassal B, Matthews L, Viteri G, Gong C, Lorente P, Fabregat A, Sidiropoulos K, Cook J, Gillespie M, Haw R, Loney F, May B, Milacic M, Rothfels K, Sevilla C, Shamovsky V, Shorser S, Varusai T, Weiser J, Wu G, et al. 2020. The reactome pathway knowledgebase *Nucleic Acids Res* **48**:D498-D503. DOI: <https://doi.org/10.1093/nar/gkz1031>, PMID: 31691815

Sahni N, Yi S, Taipale M, Fuxman Bass JI, Coulombe-Huntington J, Yang F, Peng J, Weile J, Karras GI, Wang Y, Kovacs IA, Kamburov A, Krykbaeva I, Lam MH, Tucker G, Khurana V, Sharma A, Liu YY, Yachie N, Zhong Q, et al. 2015. Widespread macromolecular interaction perturbations in human genetic disorders *Cell* **161**:647-660. DOI: <https://doi.org/10.1016/j.cell.2015.04.013>, PMID: 25910212

Li X, Cai H, Wang X, Ao L, Guo Y, He J, Gu Y, Qi L, Guan Q, Lin X, Guo Z. 2019. A rank-based algorithm of differential expression analysis for small cell line data with statistical control *Brief Bioinform* **20**:482-491. DOI: <https://doi.org/10.1093/bib/bbx135>, PMID: 29040359

Wilkerson MD, Hayes DN. 2010. ConsensusClusterPlus: a class discovery tool with confidence assessments and item tracking *Bioinformatics* **26**:1572-1573. DOI: <https://doi.org/10.1093/bioinformatics/btq170>, PMID: 20427518

Wilkerson MD, Hayes DN. 2010. ConsensusClusterPlus: a class discovery tool with confidence assessments and item tracking *Bioinformatics* **26**:1572-1573. DOI: <https://doi.org/10.1093/bioinformatics/btq170>, PMID:

Șenbabaoğlu Y, Michailidis G, Li JZ. 2014. Critical limitations of consensus clustering in class discovery *Scientific Reports* **4**:6207-6207. DOI: <https://doi.org/10.1038/srep06207>, PMID: 25158761

Verhaak RG, Hoadley KA, Purdom E, Wang V, Qi Y, Wilkerson MD, Miller CR, Ding L, Golub T, Mesirov JP, Alexe G, Lawrence M, O'Kelly M, Tamayo P, Weir BA, Gabriel S, Winckler W, Gupta S, Jakkula L, Feiler HS, et al. 2010. Integrated genomic analysis identifies clinically relevant subtypes of glioblastoma characterized by abnormalities in PDGFRA, IDH1, EGFR, and NF1 *Cancer Cell* **17**:98-110. DOI: <https://doi.org/10.1016/j.ccr.2009.12.020>, PMID: 20129251

Tusher VG, Tibshirani R, Chu G. 2001. Significance analysis of microarrays applied to the ionizing radiation response *Proc Natl Acad Sci U S A* **98**:5116-5121. DOI: <https://doi.org/10.1073/pnas.091062498>, PMID: 11309499

Tibshirani R, Hastie T, Narasimhan B, Chu G. 2002. Diagnosis of multiple cancer types by shrunken centroids of gene expression *Proc Natl Acad Sci U S A* **99**:6567-6572. DOI: <https://doi.org/10.1073/pnas.082099299>, PMID: 12011421

Marisa L, de Reynies A, Duval A, Selves J, Gaub MP, Vescovo L, Etienne-Grimaldi MC, Schiappa R, Guenot D, Ayadi M, Kirzin S, Chazal M, Flejou JF, Benchimol D, Berger A, Lagarde A, Pencreach E, Piard F, Elias D, Parc Y, et al. 2013. Gene expression classification of colon cancer into molecular subtypes: characterization, validation, and prognostic value *PLoS Med* **10**:e1001453. DOI: <https://doi.org/10.1371/journal.pmed.1001453>, PMID: 23700391

Yu G, Wang LG, Han Y, He QY. 2012. clusterProfiler: an R package for comparing biological themes among gene clusters *OMICS* **16**:284-287. DOI: <https://doi.org/10.1089/omi.2011.0118>, PMID: 22455463

Hanzelmann S, Castelo R, Guinney J. 2013. GSVA: gene set variation analysis for microarray and RNA-seq data *BMC Bioinformatics* **14**:7. DOI: <https://doi.org/10.1186/1471-2105-14-7>, PMID: 23323831

Karasaki T, Nagayama K, Kuwano H, Nitadori JI, Sato M, Anraku M, Hosoi A, Matsushita H, Morishita Y, Kashiwabara K, Takazawa M, Ohara O, Kakimi K, Nakajima J. 2017. An Immunogram for the Cancer-Immunity Cycle: Towards Personalized Immunotherapy of Lung Cancer *J Thorac Oncol* **12**:791-803. DOI: <https://doi.org/10.1016/j.jtho.2017.01.005>, PMID: 28088513

Ayers M, Lunceford J, Nebozhyn M, Murphy E, Loboda A, Kaufman DR, Albright A, Cheng JD, Kang SP, Shankaran V, Piha-Paul SA, Yearley J, Seiwert TY, Ribas A, McClanahan TK. 2017. IFN-gamma-related mRNA profile predicts clinical response to PD-1 blockade *J Clin Invest* **127**:2930-2940. DOI: <https://doi.org/10.1172/JCI91190>, PMID: 28650338

Jiang P, Gu S, Pan D, Fu J, Sahu A, Hu X, Li Z, Traugh N, Bu X, Li B, Liu J, Freeman GJ, Brown MA, Wucherpfennig KW, Liu XS. 2018. Signatures of T cell dysfunction and exclusion predict cancer immunotherapy response *Nat Med* **24**:1550-1558. DOI: <https://doi.org/10.1038/s41591-018-0136-1>, PMID: 30127393

Charoentong P, Finotello F, Angelova M, Mayer C, Efremova M, Rieder D, Hackl H, Trajanoski Z. 2017. Pan-cancer Immunogenomic Analyses Reveal Genotype-Immunophenotype Relationships and Predictors of Response to Checkpoint Blockade *Cell Rep* **18**:248-262. DOI: <https://doi.org/10.1016/j.celrep.2016.12.019>, PMID: 28052254

Lawrence MS, Stojanov P, Polak P, Kryukov GV, Cibulskis K, Sivachenko A, Carter SL, Stewart C, Mermel CH, Roberts SA, Kiezun A, Hammerman PS, McKenna A, Drier Y, Zou L, Ramos AH, Pugh TJ, Stransky N, Helman E, Kim J, et al. 2013. Mutational heterogeneity in cancer and the search for new cancer-associated genes *Nature* **499**:214-218. DOI: <https://doi.org/10.1038/nature12213>, PMID: 23770567

Mermel CH, Schumacher SE, Hill B, Meyerson ML, Beroukhim R, Getz G. 2011. GISTIC2.0 facilitates sensitive and confident localization of the targets of focal somatic copy-number alteration in human cancers *Genome Biol* **12**:R41. DOI: <https://doi.org/10.1186/gb-2011-12-4-r41>, PMID: 21527027

Liu Z, Zhang Y, Shi C, Zhou X, Xu K, Jiao D, Sun Z, Han X. 2021. A novel immune classification reveals distinct immune escape mechanism and genomic alterations: implications for immunotherapy in hepatocellular carcinoma *J Transl Med* **19**:5. DOI: <https://doi.org/10.1186/s12967-020-02697-y>, PMID: 33407585

Yang C, Huang X, Li Y, Chen J, Lv Y, Dai S. 2021. Prognosis and personalized treatment prediction in TP53-mutant hepatocellular carcinoma: an in silico strategy towards precision oncology *Brief Bioinform* **22**. DOI: <https://doi.org/10.1093/bib/bbaa164>, PMID: 32789496

Quon G, Haider S, Deshwar AG, Cui A, Boutros PC, Morris Q. 2013. Computational purification of individual tumor gene expression profiles leads to significant improvements in prognostic prediction *Genome Med* **5**:29. DOI: <https://doi.org/10.1186/gm433>, PMID: 23537167

Yang C, Chen J, Li Y, Huang X, Liu Z, Wang J, Jiang H, Qin W, Lv Y, Wang H, Wang C. 2021. Exploring subclass-specific therapeutic agents for hepatocellular carcinoma by informatics-guided drug screen *Brief Bioinform* **22**. DOI: <https://doi.org/10.1093/bib/bbaa295>, PMID: 33167027

Geeleher P, Cox N, Huang RS. 2014. pRRophetic: an R package for prediction of clinical chemotherapeutic response from tumor gene expression levels *PLoS One* **9**:e107468. DOI: <https://doi.org/10.1371/journal.pone.0107468>, PMID: 25229481
